# Gene expression analysis of TIL rich HPV-driven head and neck tumors reveals a distinct B-cell signature when compared to HPV independent tumors

**DOI:** 10.18632/oncotarget.10788

**Published:** 2016-07-22

**Authors:** Oliver Wood, Jeongmin Woo, Gregory Seumois, Natalia Savelyeva, Katy J. McCann, Divya Singh, Terry Jones, Lailah Peel, Michael S. Breen, Matthew Ward, Eva Garrido Martin, Tilman Sanchez-Elsner, Gareth Thomas, Pandurangan Vijayanand, Christopher H. Woelk, Emma King, Christian Ottensmeier, for the SPARC Consortium

**Affiliations:** ^1^ Faculty of Medicine, University of Southampton & University Hospital Southampton, Southampton, UK; ^2^ Department of Molecular and Clinical Cancer Medicine, University of Liverpool, Liverpool, UK; ^3^ La Jolla Institute for Allergy & Immunology, La Jolla, CA, USA

**Keywords:** head and neck squamous cell carcinoma, human papilloma virus, tumor-infiltrating lymphocyte, RNA-Sequencing, transcriptome

## Abstract

Human papilloma virus (HPV)-associated head and neck squamous cell carcinoma (HNSCC) has a better prognosis than it's HPV negative (HPV(−)) counterpart. This may be due to the higher numbers of tumor-infiltrating lymphocytes (TILs) in HPV positive (HPV(+)) tumors. RNA-Sequencing (RNA-Seq) was used to evaluate whether the differences in clinical behaviour simply reflect a numerical difference in TILs or whether there is a fundamental behavioural difference between TILs in these two settings. Thirty-nine HNSCC tumors were scored for TIL density by immunohistochemistry. After the removal of 16 TIL_low_ tumors, RNA-Seq analysis was performed on 23 TIL_high/med_ tumors (HPV(+) n=10 and HPV(−) n=13). Using EdgeR, differentially expressed genes (DEG) were identified. Immune subset analysis was performed using Functional Analysis of Individual RNA-Seq/ Microarray Expression (FAIME) and immune gene RNA transcript count analysis. In total, 1,634 DEGs were identified, with a dominant immune signature observed in HPV(+) tumors. After normalizing the expression profiles to account for differences in B- and T-cell number, 437 significantly DEGs remained. A B-cell associated signature distinguished HPV(+) from HPV(−) tumors, and included the DEGs *CD200, GGA2, ADAM28, STAG3, SPIB, VCAM1, BCL2* and *ICOSLG*; the immune signal relative to T-cells was qualitatively similar between TILs of both tumor cohorts. Our findings were validated and confirmed in two independent cohorts using TCGA data and tumor-infiltrating B-cells from additional HPV(+) HNSCC patients. A B-cell associated signal segregated tumors relative to HPV status. Our data suggests that the role of B-cells in the adaptive immune response to HPV(+) HNSCC requires re-assessment.

## INTRODUCTION

Head and neck squamous cell carcinoma (HNSCC) accounts for 6% of all cancers, with ~650,000 new cases reported and 350,000 HNSCC-related deaths per year worldwide [1, 2]. Historically, the risk factors for HNSCC have been smoking and alcohol [3]. However, changes in social behaviour have led to an increase in human papilloma virus (HPV)-associated HNSCC [4]. The incidence of HPV-associated HNSCC is approximately 30% [5–7], of which the majority are caused by HPV16 within the anatomical location oropharynx, which includes the base of tongue and tonsil [6]. HPV positive (HPV(+)) patients have a significantly better prognosis than HPV negative (HPV(−)) patients, with the 3- and 5-year survival at 84% and 62% for HPV(+) patients compared to 57% and 26% for HPV(−) patients, respectively [8].

A high number of tumor-infiltrating lymphocytes (TILs) is linked to good prognosis in many solid tumors, including HPV(+) HNSCC [9]. More recent analyses of The Cancer Genome Atlas (TCGA) data demonstrate that the effect is mediated by CD8^+^ GZMA^+^ PRF1^+^ T-cells [10, 11]. In HPV(+) disease, the persistent viral oncoproteins E6 and E7 cause the malignant phenotype, while the immunological visibility of these two proteins contributes to the infiltration of the tumor by T-cells [12]. By contrast, HPV(−) tumors are considered a separate disease entity and are driven by heterogeneous genetic events [11, 13]. Differential gene expression profiling comparing HPV(+) and HPV(−) tumors using microarray, RNA-Sequencing (RNA-Seq) and RT-PCR have led to an improved understanding of the events associated with cellular transformation and oncogenesis [14–17]. Thurlow *et al*. have used spectral clustering and gene ontology (GO) analysis to identify discrete gene expression patterns that linked to patient outcome, which involved the genes of adaptive and innate immunity [17]. However, the underlying biology of TILs has not been addressed despite their clear link to survival [7, 11, 14–16, 18, 19].

Our aim was to evaluate whether transcriptome analysis would identify characteristics that could differentiate TILs in HPV(+) from those in HPV(−) tumors. We established and optimized sample collection from a cohort of consecutive patients under our care that were undergoing surgery for HNSCC. Tumor samples were processed according to controlled standard operating procedures and evaluated both morphologically and by immunohistochemistry (IHC) on the one hand, and by RNA-Seq to determine whole tumor transcriptomes on the other. We demonstrated that after correction for the number of immune cells infiltrating the tumors, the T-cell signature between HPV(+) and HPV(−) tumors was very similar. By contrast, B-cell associated genes emerged as differentially expressed. High expression of these genes proved to be a distinguishing immunological feature of HPV(+) HNSCC, suggesting a fundamental biological difference in adaptive immune responses against virally driven versus virus-independent tumors. We were able to verify our findings in a large publicly available dataset from TCGA (HNSC) and also by RT-qPCR of the key DEGs.

## RESULTS

### Prognostic effect of TIL density

**Figure 1:**
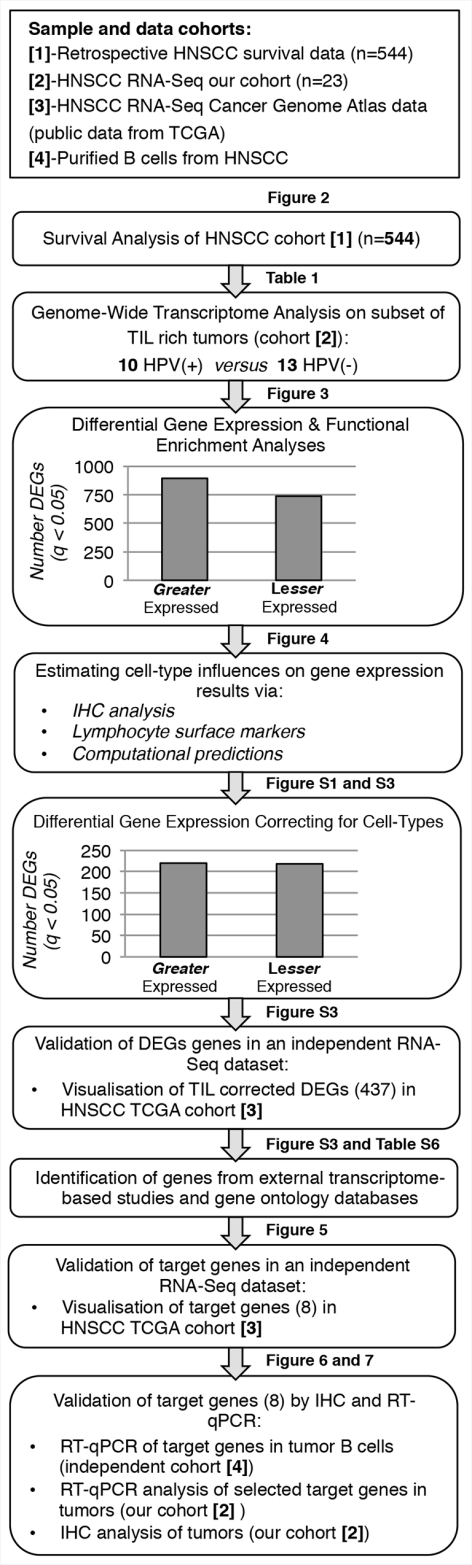
Overview of experimental procedures and analysis used to evaluate TIL Schematic representation of the multi-step analysis performed to understand the features of tumor-infiltrating immune cells in patients with HPV(+) and HPV(−) HNSCC.

We had previously demonstrated that in HPV(+) HNSCC TIL density correlates with outcome [7]. From this starting point we undertook a multi-step analysis to understand the features of tumor-infiltrating immune cells in patients with both HPV(+) and HPV(−) HNSCC (Figure 1).

**Figure 2:**
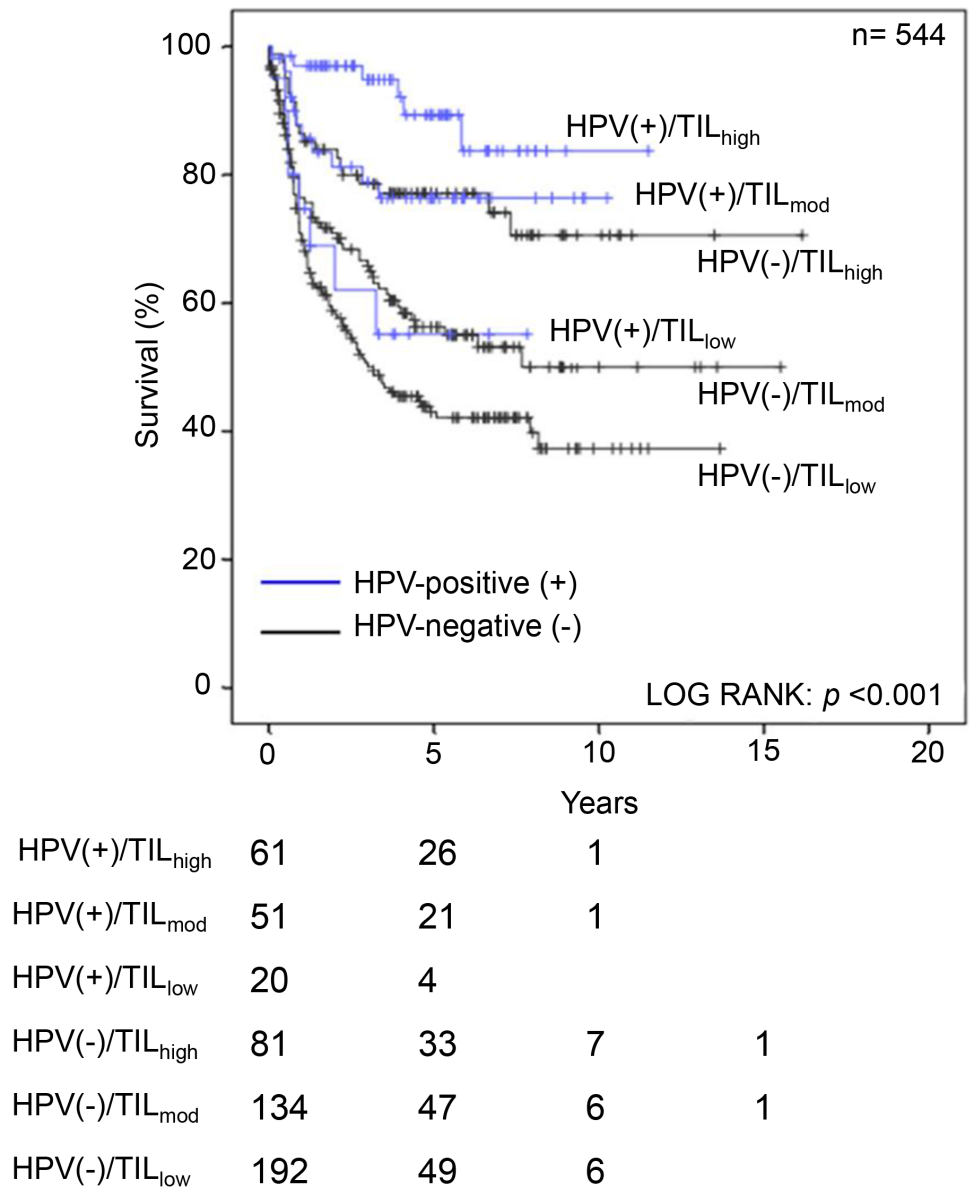
Kaplan-Meier curves for HNSCC mortality stratified according to HPV status and TIL density Survival of a retrospective cohort of HNSCC patients (n=544) with respect to HPV status and the density of immune cell infiltrate was assessed. TIL density predicts for outcome in both the HPV(+) and HPV(−) patients; log-rank test, p<0.001.

Using the same methodology as previously [7], we extended our cohort (n=544) to include HPV(−) HNSCC. As in HPV(+) tumors, TIL status stratified for outcome in HPV(−) HNSCC (Figure 2). Furthermore, HPV(+) TIL_high/mod_ patients had significantly better survival compared to HPV(−) TIL_high/mod_ patients (Figure 2, log rank p<0.001). In order to better understand this survival difference, we examined the transcriptome associated with TILs in a prospective cohort (n=39), patient demographics, tumor characteristics and tumor sampling/ processing information for the HPV(+) and HPV(−) patient cohorts are shown in Table 1. As our interest was in understanding immune cells, we focussed on TIL rich (TIL_high/mod_) tumors and excluded the 16 TIL_low_ cases from our analyses. Of the 23 TIL_high/mod_ cases, 10 were HPV(+) and 13 were HPV(−). The clinical and histological descriptors were distributed as expected (Table 1); HPV(+) tumors were located in the oropharynx of non-smoking patients, HPV(−) tumors were located in the larynx (n=4), oral cavity (n=5) and oropharynx (n=4). The clinical classification of HPV status was determined by routine IHC against p16 and mapped appropriately to the expression of E6 and E7, as detected by RNA-Seq (Table 1).

**Table 1:** Clinical and histopathological information for HPV(+) and HPV(−) HNSCC patients

Variable	Description	HPV(+)	HPV(−)	*p*-value	Test^≠^
		(n=10)	(n=13)		
Gender	% Male	90.00	76.92	6.04E-01	*a*
Age	Mean	56.90 ± 8.66	63.77 ± 15.85	2.32E-01	*b*
Collection site	Liverpool	1	0		
	Poole	2	6	2.81E-01	*a*
	Southampton	7	7		
Sequencing Batch	1	3	6		
	2	7	5	3.92E-01	*a*
	3	0	2		
RIN^*^	Mean	8.51 ± 0.80	8.51 ± 1.01	7.56E-01	*c*
Smoking	Non smoker	10	6		
	Smoker	0	4	2.56E-02	*a*
	Heavy smoker	0	3		
Alcohol	Non drinker	5	3		
	Moderate drinker	4	9	4.02E-01	*a*
	Heavy drinker	1	1		
Tumor site	Larynx	0	4		
	Oral	0	5	1.60E-03	*a*
	Oropharynx	10	4		
White blood cells	Mean	9.47 ± 2.23	8.52 ± 1.92	2.86E-01	*b*
Lymphocytes	Mean	1.82 ± 0.60	1.77 ± 0.81	5.76E-01	*c*
Staging	I	0	3		
	II	1	0	3.48E-01	*a*
	III	2	3		
	IV	7	7		
TIL status	High	8	4	3.61E-02	*a*
	Medium	2	9		
Pattern of invasion	Cohesive	10	6	7.49E-03	*a*
	Discohesive	0	7		
Differentiation	Poor	9	2		
	Moderate	1	9	8.85E-04	*a*
	Well	0	2		
Smooth muscle actin	Low	10	9		
	Moderate	0	2	2.37E-01	*a*
	High	0	2		
Tumor cell	%	68.00 ± 20.58	65.38 ± 21.06	7.69E-01	*B*
E6 expression	Mean	4.57 ± 1.11	−4.36 ± 0.57	1.75E-06	*C*
E7 expression	Mean	5.07 ± 1.30	−4.36 ± 0.57	1.75E-06	*C*

* RIN; RNA integrity number

≠ *P*-values were obtained from Fisher's exact test (*a*) for categorical variables. A two sample t-test (*b*) was performed for numerical variables with normal distribution (Shapiro-Wilk test, P≥0.05). A Wilcoxon rank sum test (*c*) was performed for numerical variables with non-normal distribution (Shapiro-Wilk test, P<0.05). Statistically significant *p*-values (<0.05) are indicated in bold.

### Gene expression analysis of HPV(+) and HPV(−) tumors

**Figure 3:**
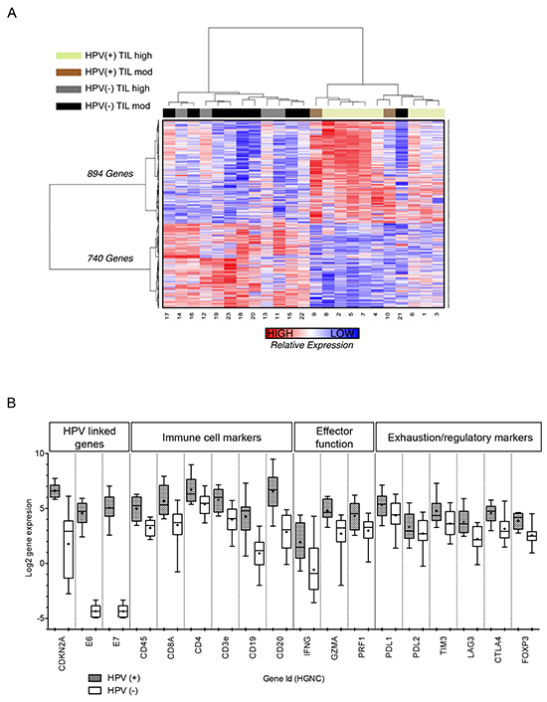
Differentially expressed genes between HPV(+) and HPV(−) tumors **A.** a heatmap to illustrate the DEGs between HPV(+) and HPV(−) tumors; each row represents the z-score of normalized gene expression values for a given gene; each column represents the gene expression for a given tumor. Z-scores are calculated from the average gene expression, plus standard deviation, in all tumors for a given gene, this enables the relative comparison of gene expression between tumors; the scale of z-score is shown: red shading denotes greater gene expression, blue shading denotes lower gene expression. Hierarchical clustering of genes and tumors based on their expression profile is reflected in the dendrograms to the left and the top of the heatmap, respectively, and was performed by calculating distance using the Pearson's correlation metric and then clustering distance using the ward linkage method^*^. **B.** the expression of key genes associated with HPV, immune cell markers, immune effector function and immune exhaustion/regulation are displayed for HPV(+) and HPV(−) tumors as box plots (min/max) with the + representing the mean. A greater expression of immune associated genes is observed in HPV(+) tumors^**^. ^*^Unsupervised clustering of gene expression data was normalized using the TMM method followed by variance stabilizing transformation of the TMM normalized data. ^**^Gene expression data from the normalized transcript counts; data was normalized using the TMM method followed by variance stabilizing transformation of the TMM normalized data.

Following RNA-Seq analysis, 1,634 genes were identified as significantly differentially expressed (*q*-value <0.05) between the HPV(+) and HPV(−) tumors (Figure 3A and Supplementary Table S1). Of these genes, 894 were expressed to a greater extent and 740 to a lesser extent in HPV(+) compared to HPV(−) tumors. These gene expression differences segregated HPV(+) and HPV(−) tumors in all except one HPV(−) subject (patient 21), whose tumor clustered within the HPV(+) cohort (Figure 3A). On histological review, this patient had a basaloid SCC, which is a rare and clinically distinct HNSCC subform. We therefore removed this case from further evaluation.

Gene expression of the HPV associated genes *CDKN2A* (*p16*), *E6* and *E7* were as expected between HPV(+) and HPV(−) tumors (Figure 3B). We also found differences in the expression of genes associated with ‘immune cell markers’ between HPV(+) and HPV(−) tumors (Figure 3B); expression of these genes was greater in HPV(+) tumors. Similarly, the expression of *GZMA*, *IFNG* and *CDNK2A* and key genes that link to T-cell activation and exhaustion, such as *CTLA4*, *PD1* and *HAVCR2* (encoding TIM3), were all increased in HPV(+) compared to HPV(−) tumors.

#### GO and pathway analysis

GO and pathway analysis was performed to understand the biological significance of the 1,634 DEGs. GO terms that were significantly over-represented for DEGs were identified using CPDB [20]; GO analysis was performed independently for those genes expressed to a greater extent and for genes expressed to a lesser extent in HPV(+) compared to HPV(−) tumors. Full details of the GO terms, including the specific genes, number and percentage of DEGs associated with each GO term, are presented in Supplementary Tables S2 and Supplementary S3 respectively.

Our data revealed that those genes with greater expression in the HPV(+) cohort were predominantly associated with an immune reaction (e.g., adaptive immune response*, GO:0002250;* lymphocyte activation*, GO:0046649;* positive regulation of immune system process*, GO:0002684;* B-cell activation, GO:0042113), whereas those expressed to a lesser extent were associated with cellular processes involved in tissue development, (*GO:0009888*), keratinization (*GO:0031424*) and cell differentiation (*GO:0030154*). Specifically, there was greater expression of genes associated with the adaptive immune system, including T-cells (CD4^+^ and CD8^+^) and B-cell receptor signalling pathways, as well as NK-cell-mediated cytotoxicity (Supplementary Table S2).

Those genes expressed to a lesser extent represented different biological processes, including extracellular matrix organisation, collagen formation, beta1 integrin cell surface interactions and alpha6 beta4 integrin-ligand interactions (Supplementary Table S3). The biological pathways (e.g., *KEGG*) over-represented for DEGs mirrored the results of GO analysis displaying an enriched number of genes linked to immunological signalling pathways in greater expressed genes (Supplementary Tables S4) and pathways linked to extracellular matrix organisation and collagen formation in genes expressed to a lesser extent (Supplementary Tables S5).

#### Quantification of TILs

GO and pathway analysis indicated that the biological processes related to the immune system and specifically to B and T-cells were over-represented for genes expressed to a greater extent in HPV(+) tumors (Figure 3B and Supplementary Tables S2 and S4). However, it was not clear whether these B- and T-cell immune-related terms identified by GO and pathway analysis were simply the result of numerical differences in lymphocytes between HPV(+) and HPV(−) tumors or the result of differences on a per cell basis in the transcriptional signature.

**Figure 4:**
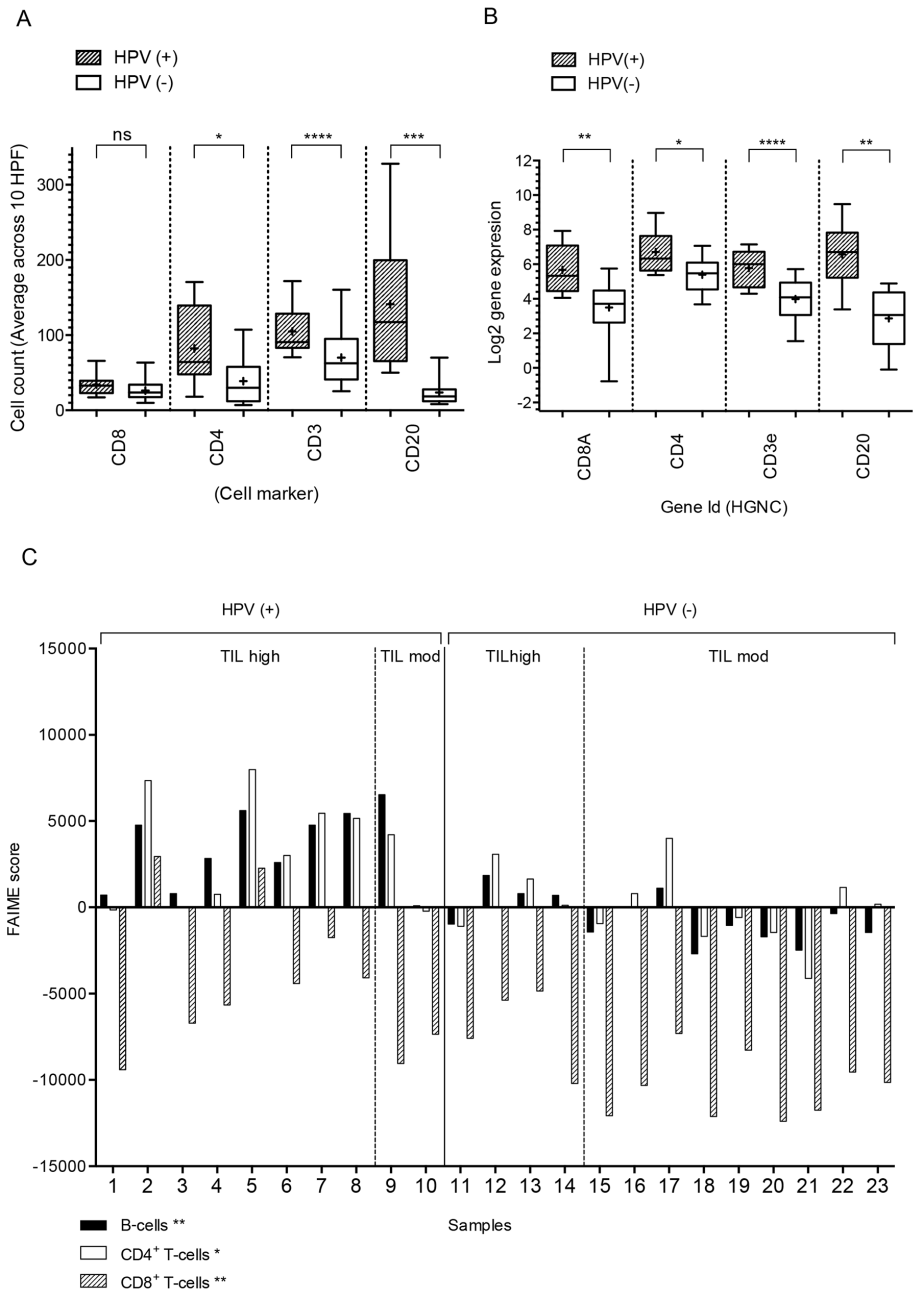
Immune cell subset analysis of HPV(+) and HPV(−) tumors **A.** The distribution of CD4, CD8, CD3 and CD20-expressing cells in HPV(+) and HPV(−) tumors as detected by IHC; cell counts are given as a mean of 10 high-power fields. **B.** Gene expression of *CD4*, *CD8A*, *CD3E* and *CD20* of HPV(+) and HPV(−) tumors displayed as box plots (min/max) with the + representing the mean^*^. Differences in TIL density between HPV(+) and HPV(−) tumors are observed both by gene expression profiling and IHC analysis. **C.** the FAIME score of lymphocytes in HPV(+) and HPV(−) tumors; the difference in distribution of specific cell subsets based on ranked gene expression is shown. *Asterisks* in column labels indicate a significance level of two-sample *t*-test comparisons of FAIME scores between HPV(+) and HPV(−) tumors: ^*^P <0.05 and ^**^P <0.01). ^*^Gene expression data from the normalized transcript counts; data was normalized using the TMM method followed by variance stabilizing transformation of the TMM normalized data.

To be able to address this question we compared three approaches to quantifying tumor-infiltrating lymphocytes: IHC for CD4, CD8, CD20 and CD3 followed by manual counting of 10 high-power fields (Figure 4A), gene RNA transcript levels (log2 normalised) for lymphocyte cell surface markers (Figure 4B) and computational evaluation using FAIME to measure the size of lymphocyte subsets in each sample (Figure 4C). A significant difference in cell number was observed between HPV(+) and HPV(−) tumors for the cell markers CD4, CD20 and CD3 (P ≤ 0.05) but not CD8 (ns) by IHC. Gene RNA transcript levels were significantly different for all cell markers (*CD4*, *CD20*, *CD3e* and *CD8A*) (Figure 4B). Spearman correlation analysis between IHC and gene expression for the cell markers CD8 (R^2^=0.76), CD3 (R^2^=0.52) and CD19 (R^2^=0.47) is shown in Supplementary Figure S1A, B and C respectively. FAIME analysis uses gene expression biomarkers (Supplementary Table S6) to score the size of each lymphocyte subset; this score was significantly different between the HPV(+) and HPV(−) tumors for B-cells (*q*-value =1.35E-03), CD4^+^ T-cells (*q*-value =2.66E-02) and CD8^+^ T-cells (*q*-value =9.64E-03). FAIME scores indicated that the number of cells belonging to these lymphocyte subsets was higher in HPV(+) tumors compared to HPV(−) tumors.

Both molecular analyses demonstrated that the numbers of B-cells and CD4^+^ and CD8^+^ T-cells were higher in HPV(+) tumors compared to HPV(−) tumors, this was confirmed by the ‘gold standard’ assessment by IHC for B-cells, CD3^+^ T-cells and CD4^+^ T-cells (P ≤ 0.05). A significant difference was not observed by IHC for CD8^+^ T-cells, however a trend towards higher numbers in HPV(+) was observed (Figure 4A).

### Analysis of gene expression data following correction for numerical differences in TILs

The global gene expression data were next corrected for TIL number using the gene expression of *CD19* (pan B-cell marker, CD20 was used only for IHC comparison), *CD4* and *CD8A* in each sample as a covariate. When correcting both HPV(+) and HPV(−) cohorts in this way, genes co-ordinately expressed in lymphocyte subsets were no longer differentially expressed; as a result the number of DEGs dropped from 1,634 to 437 (Supplementary Table S7). As expected, there was a large overlap in DEGs between the initial uncorrected and the TIL corrected data sets (Supplementary Figure S2). The TIL corrected dataset was next subject to GO and pathway analysis.

#### GO and pathway analysis of TIL corrected data

GO and pathway analysis was again performed independently for genes expressed to a greater extent (n=219; Supplementary Table S8) and a lesser extent (n=218; Supplementary Table S9) in HPV(+) compared to HPV(−) tumors. Consistent with the FAIME analysis, the vast majority of immune and lymphocyte-related terms were no longer over-represented in HPV(+) tumors. This included markers of T-cell effector function (e.g., *IFNG, GZMB* and *PRF1*; Figure 3B), which prior to TIL correction were all over-represented in the HPV(+) tumors. Pathway analysis also confirmed the loss of immune-related signalling pathways (Supplementary Table S10).

Immune GO terms that remained following correction for numerical differences in TIL were B-cell activation *(GO:0042113)*, which included *BCL2*, *VCAM1* and *ICOSLG*, with a greater expression in HPV(+) compared to HVP(−) tumors (Supplementary Table S8). The surviving non-immune GO terms and biological pathways (Supplementary Table S8 and S10) over-represented in HPV(+) tumors were associated with cell cycle (*GO:0007049*), cell phase transition (*GO:0044770*) and chromosome organisation (*GO:0051276*).

The loss of T-cell and the majority of B-cell-related GO terms following TIL correction of gene expression data indicated that gene expression differences between HPV(+) and HPV(−) tumors largely resulted from numerical differences in these cells types. To determine if any differences in lymphocyte gene expression between the HPV(+) and HPV(−) tumor cohorts were retained following TIL correction, the DEGs identified after correction were overlapped with the lymphocyte-specific marker genes used to determine the cell proportions in the FAIME analysis (CD19^+^ B-cell genes and CD8^+^ and CD4^+^ T-cell genes, Supplementary Table S6 [21–26]). The majority of the surviving signals were associated with the following B-cell associated genes: *GGA2*, *SPIB*, CD200, *ADAM28* as well as *STAG3*, which was not previously known to be a B-cell gene. A single CD8-associated gene (*CD8B*) also survived correction (Supplementary Figure S3).

### Validation of findings using TCGA RNA-Seq data

We next assessed the expression of the identified DEGs (n=437) including the 8 B-cell-related genes *GGA2*, *SPIB*, CD200, *ADAM28, BCL2*, *VCAM1, ICOSLG* and *STAG3*, in an independent HNSCC dataset from TCGA (TCGA HNSC HiSeqV2 2015-02-24 data source outlined in methods) [11].

**Figure 5:**
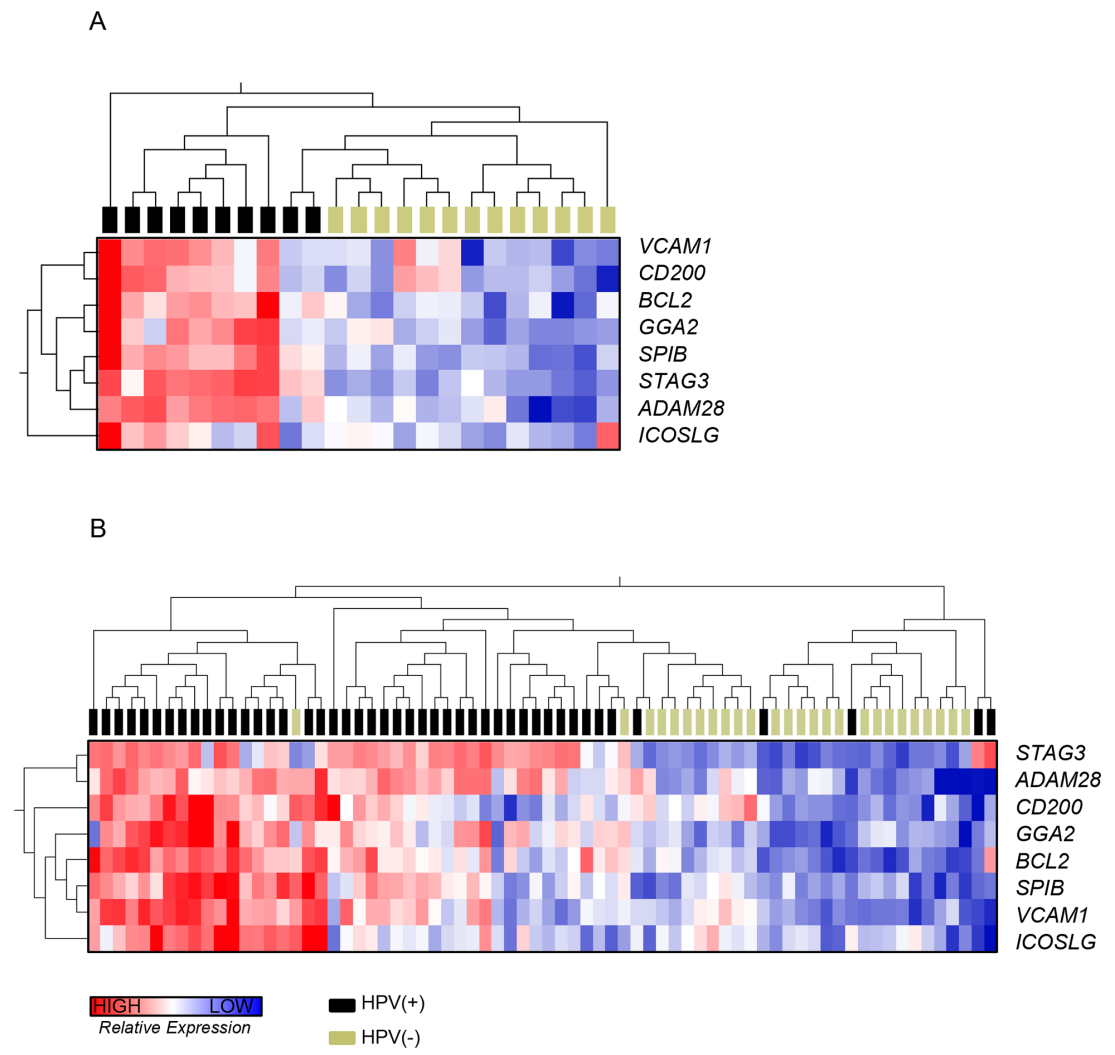
Expression of B-cell-associated genes by RNA-Seq Heatmaps* to illustrate gene expression of the identified B-cell-associated genes between HPV(+) and (−) tumors: *GGA2*, *SPIB*, CD200, *STAG3, ADAM28, BCL2*, *VCAM1* and *ICOSLG*. **A.**, a heatmap of our HNSCC dataset (HPV(+) n=10 and HPV(−) n=13). **B.**, a heatmap of the TCGA HNSCC dataset (HPV(+) n=46 and HPV(−) n=26); publically available data from anatomically matched tumors arising in the oropharynx, tonsil and base of tongue. In both datasets, tumors cluster according to HPV status, with a greater expression of the identified B-cell-associated genes in HPV(+) tumors. ^*^Unsupervised clustering of gene expression data was normalized using the TMM method followed by variance stabilizing transformation of the TMM normalized data. Each row represents normalized gene expression values for a given gene; each column represents the gene expression for a given tumor: red shading denotes greater gene expression, blue shading denotes lower gene expression. Hierarchical clustering of genes and tumors based on their expression profile is reflected in the dendrograms to the left and the top of the heatmap, respectively, and was performed by calculating distance using the Pearson's correlation metric and then clustering distance using the ward linkage method.

Analysis of TCGA data allowed us to address whether the DEGs might be the result of anatomical bias: since HPV(+) tumors predominantly arise in specific anatomical locations (tonsil, base of tongue and oropharynx) these might per se contribute to DEGs. Hence we identified 72 cases (46 HPV(+) and 26 HPV(−)) from anatomically matched locations (tonsil, base of tongue and oropharynx). Visualisation of the 8-gene signature (*GGA2*, *SPIB*, CD200, *ADAM28, BCL2*, *VCAM1, ICOSLG* and *STAG3)* identified from our cases in a hierarchically clustered heatmap (Figure 5A) together with the 72 TCGA cases (Figure 5B) demonstrated the clustering of tumors according to HPV status, with a greater gene expression in HPV(+) compared to HPV(−) tumors. These data confirmed that anatomical bias was not the reason for the B-cell-associated differences in gene expression. Unsupervised hierarchical clustering of all 437 TIL corrected DEGs for our own dataset and TCGA data is shown in Supplementary Figure S4.

### Validation of RNA-seq data by RT-qPCR and IHC

**Figure 6:**
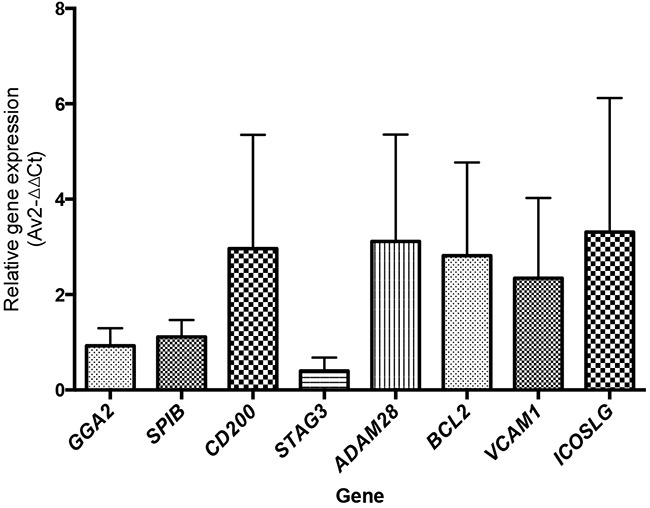
Relative expression of B-cell-associated genes by RT-qPCR The average relative gene expression of B-cell-associated genes was measured by RT-qPCR^*^ in HPV(+) tumors. The expression of the B-cell-associated genes *GGA2*, *SPIB*, *CD200*, *STAG3*, *ADAM28*, *BCL2*, *VCAM1* and *ICOSLG* was confirmed in B-cells sorted from an independent cohort of HPV(+) tumors (n=6). ^*^Relative gene expression by RT-qPCR, calculated using the comparative Ct method with *Actin* as the control gene (2-ΔΔCt method) (23).

We confirmed our findings with RT-qPCR, identifying the expression of the genes *GGA2*, *SPIB*, CD200, *STAG3, ADAM28, BCL2*, *VCAM1* and *ICOSLG* in B-cells isolated from an independent HPV(+) HNSCC tumor cohort (n=6) (Figure 6). In addition to this, RT-qPCR of *CD200* and *STAG3* was carried out on the whole tumor RNA samples used for the RNA-Seq (n=8 HPV(+) and n=8 HPV(−). This showed the same trend with HPV(+) tumors compared to HPV(−) tumors having increased expression of *STAG3* and *CD200* (Supplementary Figure S5; *STAG3*, ***p<0.001 and *CD200* nsd, p=0.116). We have exhausted the material meaning additional genes and cases could not be assessed, limiting the statistical power for CD200. *STAG3*, a component of the meiosis specific cohesin complex [27] was expressed at a low level in B-cells, RT-qPCR of whole tumor tissue confirmed its differential expression between HPV(+) and HPV(−) tumors (Supplementary Figure S5).

**Figure 7:**
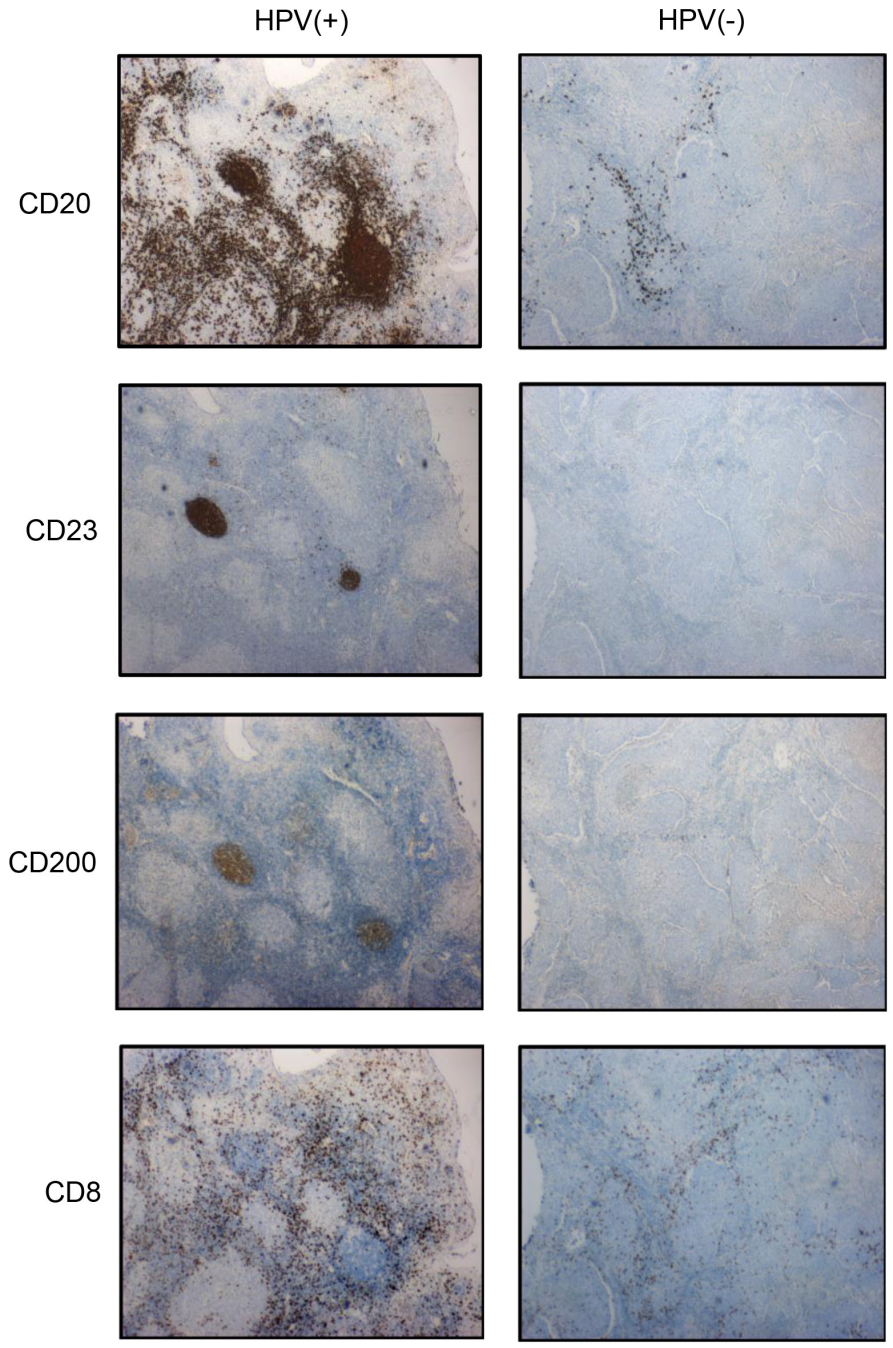
Expression of B-cell markers by IHC Cell subset anlalysis by IHC for the B-cell markers CD20, CD23 and CD200, as well as the T-cell marker CD8, was performed on sequential sections for HPV(+) (n=9) and HPV(−) (n=13) tumors; representative data is shown for one tumor from each cohort. Pseudo-follicle formation is apparent in HPV(+) tumors along with dense infiltrate of CD20^+^ B-cells. TIL density was greater in HPV(+) tumors.

IHC assessment of HPV(+) and HPV(−) tumors identified dense clusters of tumor-infiltrating B-cells in the former; Figure 7 shows representative histology for one HPV(+) and one HPV(−) tumor. The follicular morphology within HPV(+) tumors was apparent following staining for CD23, a marker of follicular B-cells. Furthermore, IHC confirmed the presence of CD200^+^ cells within and around tertiary lymphoid follicles, together with a diffuse CD8^+^ T-cell infiltrate.

### Retained non-immune gene differences between HPV(+) and HPV(−) tumor after TIL correction

GO and pathway analysis of DEGs expressed to a lesser extent in HPV(+) compared to HPV(−) tumors were largely unchanged following TIL correction of the data. There were 218 lesser expressed genes following lymphocyte correction, of which many were associated with development (skin, epidermis, epithelium, tissue, organ) and keratinization (Supplementary Figure S2 and Supplementary Table S9). In addition, lesser expressed genes associated with IL-12 and IL-6 production, the inflammatory response and the response to oxidative stress. Individual DEG's included keratin's (*KRT-10*, *14*, *16* and *17*), kallikrein-related peptidase 5, 7 and 14 (*KLK5, 7 and 14*), caspase 14 (*CASP14*), tumor necrosis factor (ligand) superfamily member 9 (*TNFSF9* or *CD137L*), thrombospondin receptor (*CD36*) and chemokine (C-C motif) ligand 19 (*CCL19*); pathway analysis of lesser expressed genes shown in Supplementary Table S11 returned no significantly over-represented pathways (q-value <0.05).

## DISCUSSION

We have previously shown that HPV(+) HNSCC patients with a dense immune cell infiltrate within the tumor have a better outcome than those with a sparse infiltrate [7] and show here that this is also the case for HPV(−) tumors. We wished to evaluate whether fine-resolution transcriptomic analysis could give insight into the biological difference in the immune infiltrate between patients with a known viral (HPV) driver, compared with those patients where virus is absent. Although differential gene expression studies comparing HPV(+) and HPV(−) tumors have been reported [11, 14–17], the question as to whether the pathogenesis of the cancer is reflected in differences in immune cells themselves remains open.

In this study we focused on TIL_high/mod_ cases from our consecutive cohort and following RNA-Seq analysis we examined DEGs. Initially, the most striking difference was the immune signature, which was significantly greater in HPV(+) tumors and hence likely reflected an immune responses to viral antigens. In contrast HPV (−) tumors revealed a prominent tissue development/ re-organisation gene signature. The molecular data mapped well onto the TIL characterization afforded by IHC, although the variability was higher when TILs were counted manually. It is likely that this is a reflection of the fact that RNA-Seq analysis uses homogenized tumor, which averages the geographical differences within the tissue.

A significant difference in TIL density determined by RNA-Seq gene transcript levels of *CD4* and *CD8A* (T-cells) and *CD19* (B-cells) remained between HPV(+) and HPV(−) patient cohorts. This was clearly demonstrated in the IHC assessment and by FAIME analysis, where in ranked order, HPV(−) TIL_high/mod_ patients had a significantly lower expression of B- and T-cell-related genes compared with HPV(+) TIL_high/mod_ patients. In order to assess the difference between HPV(+) and HPV(−) TIL enriched tumors, the data were corrected according to the number of immune cells present in the tissue as determined by RNA-Seq gene transcript levels (*CD8A*, *CD4* and *CD19*), with the aim to account for the numerical difference in immune cells.

RNA-Seq data that had been corrected for TIL number showed that the vast majority of immune-related DEGs were ‘lost’, suggesting that in both patient cohorts the lymphocytes were qualitatively similar. The T-cell immune signature, present in both HPV(+) and HPV(−) tumors, no longer showed differentially expressed genes. Thurlow *et al*. have previously demonstrated that both HPV(+) and HPV(−) cohorts can mount adaptive immune responses [19], while the association of cytolytic activity of effector cells and immunoediting of the tumor has also been reported [10]. These studies however did not correct for lymphocyte numbers; the HPV(+) tumors in which no adaptive response was detected likely represented TIL_low_ patients. We had expected to find that a viral driver would promote a distinct T-cell-driven TIL signature, but this was not observed in our data. Instead, our data demonstrate that T-cells in TIL_high/med_ tumors of different pathogeneses are transcriptomically similar, at the level of bulk population analysis.

In contrast, there was a distinct B-cell signature between HPV(+) and HPV(−) tumors after correction for TIL numbers, there was a small subset of B-cell associated genes that continued to have greater expression in HPV(+) tumors, including *GGA2*, *SPIB*, CD200*, ADAM28, BCL2*, *VCAM1* and *ICOSLG*, as determined by previously published datasets for B-cell gene signatures and the GO term (B-cell activation*, GO:0042113)* [21–26]. The expression of the differentially expressed B cell-associated genes is not unique to B cells as they can be identified in other cell types. This includes CD200, which has been found to be expressed on 1-2% of basal cell carcinoma cells [28]. However, our data on purified B-cell populations from HPV(+) HNSCC confirms the expression of the *BCL2*, *ADAM28*, *CD200*, *ICOSLG* and *SPIB* genes in B cells isolated from the tumors. We were able to confirm our DEGs in a larger, independent cohort derived from HNSCC TCGA data. We could show that the DEG are not the result of anatomical location bias of HPV(+) tumors, as the differences are maintained when anatomically matched HPV(−) and HPV(+) only are compared. Additionally, applying the TIL corrected DEG list to the TCGA confirmed the validity of our observations.

Greater expression of BCL2 by HNSCC has been proposed as a predictor of good response to chemotherapy; this is consistent with its expression in our HPV(+) cohort, a group of patients that generally respond well to treatment, including chemotherapy [29, 30]. During a normal humoral response, ICOSLG is expressed on activated B-cells within germnal centers, which are formed in follicles and are central for an antigen-specific humoral response. Histologically, all of our HPV(+) tumors had very well developed follicles/tertiary lymphoid structure [31]. Follicles within solid human tumors have previously been described in breast, cervical and non-small-cell lung carcinoma [32, 33]. In melanoma, ICOSLG is linked with increasing numbers of regulatory T-cells (T_reg)_ [34], but has not previously been described in HPV(+) HNSCC. Interestingly, when a stimulatory ICOS antibody was used in combination with anti-CTLA-4, there was a significant improvement in tumor rejection in both melanoma and colon cancer mouse models, suggesting that HPV(+) tumors expressing high levels of ICOSLG may respond better to anti-CTLA-4 therapy [35]. *SPIB* is a transcriptional activator that is specific for lymphoid cells and has previously been identified in germinal centers where it was associated with an activated B-cell phenotype [36].

Using IHC to probe protein expression of CD200, we identified CD200^+^ B-cells within and outwith of follicular structures. Given that the viral antigens E6 and E7 generate a strong B-cell response in patients with HPV(+) HNSCC [37], it is likely that the expression of the B-cell activation marker CD200 [38] is linked to a persistence of HPV-driven, tumor-derived antigens, which in turn stimulate specific B-cells in the germinal center and the tumor itself. It is also possible that the CD200^+^ B-cells resident at specific locations in the tumor vs. the follicles have a distinct phenotype and properties. These cells could be part of an inhibitory pathway: CD200R activation stimulates the differentiation of T-cells to T_reg_ [39], the numerical increase of T_reg_ in parallel with TIL number has been reported in HPV(+) HNSCC [40]. These CD200+ B cells can act through CD200R and effector mechanisms may include immunomodulatory cytokines, such as IL-10, [41] release of granzyme B [42] or other yet to be identified modes of action. The receptor is expressed on both lymphoid and myeloid cells, hence direct and indirect inhibition of T-cell immunity could be possible [43]. It must however be noted that expression of CD200 is thought to have links to anti-tumor effects by inhibiting activity of tumor-associated myeloid cells via IL-10 [44], arguing caution in targeting a molecule with potentially pleiotropic effects.

It is apparent that phenotypic differences exist between the B-cells of HPV(+) and HPV(−) tumors. Data from an HPV-driven mouse tumor model supports a reduction in tumor growth as a result of the depletion of B-cells via anti-CD20 [45]. It is tempting to speculate that anti-CD200 could provide a more targeted approach to B-cell manipulation within these tumors rather than the use of anti-CD20, which would globally remove multiple B-cell subsets. In the clinic, an anti-CD200 blocking antibody was safe and well tolerated in Phase II testing (NCT00648739), but as yet the clinical effects have not been reported. The B-cell receptor targeting therapies (ibrutinib) have efficacy on CD200^+^ cells within the B-CLL setting and potentially could be exploited [46].

This paper is the first to compare RNA-Seq data from HPV(+) and HPV(−) HNSCC, when controlled for TIL number. We have identified genes expressed both to a greater and lesser extent between tumor types. We are the first investigators to propose a correction calculation of transcriptomic signals in immune cells, to overcome the numerical imbalance between virally driven and virus-independent HNSCC. This analysis reveals that differences between the transciptome in T-cells between HPV(+) and HPV(−) HNSCC appears predominantly quantitative, but that a distinct B-cell profile exists in HPV(+) cancers. However, to truly assess qualitative differences in B- and T-cells between HPV(+) and HPV(−) tumors, these subsets must be isolated and analysed separately. Such disaggregation of tumor tissue will be the focus of future work.

## MATERIALS AND METHODS

### Study subjects and tumor processing

Following LREC approval and written informed consent, 39 consecutive HNSCC samples were obtained from patients at three centers (Southampton, n=22; Poole, n=15; Liverpool, n=2) from 2010-2012. Tumor samples were collected, following general anaesthesia but before surgical resection, and were snap frozen immediately. Cryosections (10μm) were cut and used for RNA isolation with the RNeasy Mini Kit (Qiagen Ltd., Manchester, UK). Table 1 shows the patient demographics, tumor characteristics and tumor sampling/processing information for the HPV(+) and HPV(−) patient cohorts. A retrospective cohort of 544 HNSCC patients were also used for the generation of survival data.

### Histology and immunohistochemistry

Frozen tumor sections taken immediately adjacent to the tissue analysed by RNA-Seq were stained with hematoxylin and eosin (H&E); tumors were assessed as TIL high (TIL_high_), moderate (TIL_mod_) and low (TIL_low_) by an accredited pathologist [G.J.T] as previously described [7] (Supplementary Methods S1). Formalin-fixed, paraffin-embedded (FFPE) tumor tissue blocks from the same patients were also collected and used to evaluate the cell surface marker expression of CD3, CD4, CD8, CD20, CD23 and CD200 by IHC; enumeration of CD3, CD4, CD8 and CD20 was expressed as an average of ten high-power fields [7] (Supplementary Methods S1).

### Survival data

Two HNSCC patient cohorts consisting of 137 HPV(+) and 407 HPV(−) tumors were analysed retrospectively for survival relative to TIL density and HPV status. The primary endpoint was death from HNSCC, i.e., disease-specific survival, as previously described [7].

### RNA-Sequencing and data analysis

RNA-Seq (Single end, 35 bp) was performed using the HiSeq 2000 platform (Illumina Inc., San Diego, USA) (Supplementary Methods S2). RNA-Seq data have been deposited in the Gene Expression Omnibus (GEO) at the National Center for Biotechnology Information (NCBI) under accession number GSE72536. RNA-Seq data was mapped using human genome (hg19) and TopHat (version 2.0.9), counted with HTSeq-count (version 0.5.4) [47] and differentially expressed genes (DEGs) identified with EdgeR (version 3.4.2) [48, 49]. EdgeR was also used to identify DEGs while adjusting for the covariates associated with varying proportions of lymphocyte subsets in each tumor sample, gene expression of *CD19* (B-cells) and *CD4* and *CD8A* (T-cells) were used as the covariates. Unsupervised clustering of tumors was performed following variance stabilizing transformation of trimmed mean of M-values (TMM) normalized data. DEGs between HPV(+) and HPV(−) tumors were identified with a false discovery rate (FDR) corrected *p-*value of <0.05 (i.e., *q*-value <0.05) and a fold change of >2 or <-2. A detailed description of the RNA-Seq data analysis performed can be found in the Supplementary Methods S3.

### Real-time quantitative reverse transcription PCR

Real-time quantitative reverse transcription PCR (RT-qPCR) assays were performed using Taqman® probes for golgi-associated, gamma adaptin ear containing, ARF binding protein 2 (*GGA2*), ADAM metallopeptidase domain 28 (*ADAM28*), *CD200*, Spi-B transcriptional factor (*SPIB*), stromal antigen 3 (*STAG3*), Vascular Cell Adhesion Molecule 1 (*VCAM1)*, Inducible T-Cell Co-Stimulator Ligand *(ICOSLG)* and B-Cell CLL/Lymphoma 2 (*BCL2)* and adhere to the MIQE guidelines for RT-qPCR (Supplementary Methods S4) [50]. Gene expression (RT-qPCR) of *STAG3* and *CD200* was validated in the original tumor RNA, HPV(+) n=8 and HPV(−) n=8 patient tumor samples. B-cells (CD19^+^) isolated from an independent cohort of HPV(+) tumors (n=6) using a BD FACSAria™ sorter (BD Biosciences, Oxford, UK, Supplementary Methods S4) were assessed for gene expression of *ADAM28, BCL2, CD200, GGA2, ICOSLG, SPIB, STAG3*, and *VCAM1*. RT-qPCR analysis was performed using the comparative Ct (cycle threshold) method (2^-^^ΔΔ^Ct) using *Actin* as the control gene and is defined as a normalized relative gene expression compared to a the control gene [51].

### Gene ontology and pathway analysis

GO terms associated with biological processes and biological pathways that were significantly over-represented for DEGs (*q*-value <0.05) were identified with ConsensusPathDB [20] (CPDB, release 30) using the hypergeometric test. ConsensusPathDB represents a first generation tool for functional genomics and was sufficient for the purpose of showing the change in GO terms and pathways before and after correction for the number of B- and T-cells between HPV(+) and HPV(−) tumors. GO and pathway analyses were performed for genes that were expressed (i) to a greater extent and (ii) to a lesser extent in HPV(+) compared to HPV(−) tumors.

### Molecular quantification of TILs in HPV(+) and HPV(−) tumors

The distribution and proportions of TILs were assessed at both the tumor sample and group level. At the sample level, the gene expression (RNA-Seq) and surface protein expression (IHC) of *CD3*, *CD20, CD4* and *CD8* were evaluated. In addition, “Functional Analysis of Individual RNA-Seq or Microarray Expression” (FAIME) [52] was adapted to generate a score for a large number of tissues and cell types, including B-cells and CD4^+^ and CD8^+^ T-cells (additional details in Supplementary Methods S5 and Supplementary Table S6). The FAIME score was calculated for each cell type, for each tumor and was followed by a student's t-test to assess whether the FAIME scores for a particular cell subset were significantly different (*q*-value <0.05) between the HPV(+) and HPV(−) cohorts.

### Validation of findings in TCGA data set

HNSCC RNA-Seq data (TCGA HNSC HiSeqV2 2015-02-24) was obtained from The Cancer Genome Atlas (TCGA) Genome Data Analysis Center (GDAC) Firehose website (http://gdac.broadinstitute.org/runs/stddata_2015_11_01/data/HNSC/20151101/), the RNA-Seq methodology and processing have been described by TCGA [11]. As HPV-driven cancers typically arise in the oropharynx, tonsil and base of tongue, we identified and evaluated tumors matched for these anatomical sites from TCGA. Unsupervised clustering of 46 HPV16(+) and 26 HPV(−) anatomically matched tumors from the oropharynx, tonsil and base of tongue was performed using the differential gene lists generated from our own analysis.

## SUPPLEMENTARY MATERIALS FIGURES AND TABLES
























