# Supplementary material for: Gene expression analysis of TIL rich HPV-driven head and neck tumors reveals a distinct B-cell signature when compared to HPV independent tumors
Source: Oncotarget. 2016 Jul 22;7(35):56781–97. doi: 10.18632/oncotarget.10788 (PMC5302866; doi:10.18632/oncotarget.10788)
Supplement: Supplementary file 1 [file oncotarget-07-56781-s001.pdf]

Gene expression analysis of TIL rich HPV-driven head and neck tumors reveals a distinct B-cell signature when compared to HPV independent tumors

SUPPLEMENTARY METHODS

Supplementary Methods S1, Histology and immunohistochemistry

TIL status was scored on frozen tumor sections that had been stained with H&E and viewed under low-power magnification (x2.5 objective) as described previously [1]; TIL_{high}: diffuse, present in >80% of tumor/stroma; TIL_{mod}: patchy, present in 20–80% of tumor/stroma; TIL_{low}: weak/absent, present in <20% of tumor/stroma. Data regarding the percentage tumor cells, tumor grade and pattern of invasion were also recorded. Furthermore, IHC was performed on FFPE tumor sections against CD3, CD4, CD8 and CD20 (all from Novocastra, Milton Keynes, UK). TILs were quantified using a Zeiss AxioCam MRc5 microscope (Zeiss, Cambridge, UK) and Zeiss Axiovision software (version 4.8.1.0; Zeiss) in an average of 10 high-power (x400) fields across representative areas of each tumor to allow for intratumoral heterogeneity; an average intratumoral TIL score per high-power field was calculated. Additionally, IHC was performed against the antigenic targets, CD200 (Sigma-Aldrich Company Ltd., Gillingham, UK) and CD23 (Abcam, Cambridge, UK). HPV status was evaluated by IHC against p16 (CINtec, Roche, Burgess Hill, UK) and scored as HPV(+) (>50% tumor cells positive) or HPV(−) (<50% tumor cells positive); confirmation was by evaluation of E6 and E7 RNA transcript levels from the RNA-Seq data (Table 1).

Supplementary Methods S2, RNA-Seq

RNA quality was assessed using the Agilent 2100 Bioanalyser (Agilent Technologies UK Ltd., Stockport, UK); an average RNA quality number (RIN) of 8.51±0.90 was observed across all tumor samples. Total RNA was converted into a library for sequencing on the HiSeq 2000 (Illumina Inc., San Diego, USA) using the TruSeq™ stranded mRNA Sample Preparation Kit (Illumina Inc.). Briefly, poly-A mRNA was purified from total RNA (100ng) using the Poly(A) Purist Mag Kit (Life Technologies Ltd., Paisley, UK), according to the manufacturer's instructions. The mRNA was then amplified and converted into cDNA, which was purified and used to construct libraries that were hybridized to the flow cell for single end (SE 35bp) sequencing.

Supplementary Methods S3, RNA-Seq data analysis

The quality of raw SE read data in FASTQ files was assessed and reads of low quality were trimmed or removed. SE reads were then mapped to the human genome (hg19) using TopHat (version 2.0.9) [2] and, following the removal of multi-mapping reads, converted to gene-specific read counts for 23,368 annotated genes using HTSeq-count (version 0.5.4) [3]. Non-specific filtering of count data was performed using the Bioconductor package EdgeR (version 3.4.2) [4, 5] such that genes with less than 2 read counts per million in 25% of tumor samples were excluded from further analysis. The remaining 14,528 genes were subject to normalization using the TMM method [6] to account for differences in library size from sample to sample. Unsupervised clustering of samples was performed following variance stabilizing transformation of TMM normalized data and illustrated as a heatmap.

DEGs between HPV(+) and HPV(−) groups were identified with a FDR adjusted *p*-value <0.05 (i.e., *q*-value <0.05) and a fold change of >2 or <-2 using EdgeR [4]. Fold change was calculated in EdgeR as the log2 of geometric mean of intensities; a positive and a negative fold change represents genes that were expressed to a greater or lesser extent, respectively, in HPV(+) versus HPV(−) tumors. *q*-values were obtained from differential expression test in EdgeR using the generalized linear model likelihood ratio test and adjusted for multiple testing using the Benjamini and Hochberg method to control the FDR. This package models the negative binomial distribution and implements general linear models to identify DEGs. EdgeR was also used to identify DEGs while adjusting for covariates associated with varying proportions of lymphocyte subsets in each tumor sample as reflected in the expression of CD19 (B-cells) and CD4 and CD8A (T-cells) e.g. R-script used in EdgeR for the covariate adjustment was: design <model.matrix (~adjustv_CD19+adjustv_CD4+adjustv_CD8+Group).

Supplementary Methods S4, B-cell sorting and RT-qPCR

Tumor-infiltrating B-cells were isolated from HPV(+) tumors using a combination of mechanical and enzymatic dissociation. The tumor tissue was cut into

small fragments using a scalpel. Tumor fragments were then incubated at 37°C for 15 minutes in an orbital shaker with 1-2mL RPMI 1640 medium (Gibco, Fisher Scientific UK Ltd., Loughborough, UK) containing 20 units/mL Liberase DL (Roche Diagnostics Ltd., Burgess Hill, UK) and 800 units/mL DNase I (Sigma-Aldrich Co. Ltd., Gillingham, UK). The tumor cell lysate was then passed through a 70µm filter with ice-cold RPMI 1640 medium and centrifuged at 1500rpm for 7 minutes. Cells were re-suspended in MACS buffer (1xPBS containing 2mM EDTA (pH 8.0) and 0.5% BSA) and the volume adjusted to give a concentration of <10x10^{6} cells/mL. Cells were incubated with 10µL FcR block (Miltenyi Biotec Ltd., Bisley, UK) per 100µL of cell suspension. The B-cells (CD19^{+} and CD20^{+}) were then stained with a cocktail of fluorescently conjugated antibodies (see below) at 4°C for 30 minutes: anti-CD45 FITC-conjugated (clone HI30); anti-CD4 PE-conjugated (clone RPA-T4); anti-CD3 PE-Cy7-conjugated (clone SK7); anti-CD8 PerCP-Cy5.5-conjugated (clone SK1); anti-HLA-DR APC-conjugated (clone L243); anti-CD14 APC-H7-conjugated (clone MφP9); anti-CD19 PerCP-Cy5.5-conjugated; anti-CD20 PerCP-Cy5.5-conjugated. One-thousand to 50,000 B-cells were sorted into ice-cold TRIzol LS reagent (Ambion®, Fisher Scientific UK Ltd.) at a flow rate of <2000 events/second on a BD FACSAria ™ (BD Biosciences). The time from arrival of the tumor in the laboratory to processed, sorted B-cell was <3 hours.

RNA isolation from sorted B-cells was performed using the Direct-zol™ RNA MiniPrep system (ZYMO Research Co., Irvine, USA). RT was performed on 1.5ng of RNA using SuperScript® III First-Strand Synthesis System (Invitrogen, Fisher Scientific UK Ltd.). qPCR was performed for selected genes using TaqMan Gene Expression Assays (Life Technologies Ltd.), according to the manufacturer's instructions: *GGA2* (Human Hs00370910_m1), *ADAM28* (Human Hs00248020_m1), *STAG3* (Human Hs00429370_m1), *CD200* (Human Hs01033303_m1), *SPIB* (Human Hs00162150_m1), *ICOSLG* (Hs00323621), *BCL2* (Hs01048932_g1) and *VCAM1* (Hs01003372_m1). Analysis of RT-qPCR data was performed using the comparative Ct method ($2^{-\Delta\Delta}$Ct method) using an internal control (*Actin*) and displayed as relative gene expression levels against a control sample [7]. RT-qPCR was reported in accordance with Minimum Information for Publication of Quantitative Real-Time PCR Experiments (MIQE) [8].

Supplementary Methods S5, Functional analysis of individual microarray expression (FAIME) method

The FAIME method [9] was adapted to generate a score for a large number of tissue and cell types present in each tumor sample. Marker gene sets whose expression was associated with different tissue and cell types, including lymphocyte subsets (B-cells, NK cells and CD4^{+} and CD8^{+} T-cells), were accrued from the following resources: CTen [10], IRIS, [11], HeamAtlas [12], Palmer *et al.* [13], Grigoryev *et al.* [14] and Whitney *et al*. [15]. Particular attention was paid to gene expression markers of lymphocyte origin; a marker for a particular type of lymphocyte (e.g., a B-cell) needed to be expressed in that lymphocyte as confirmed in at least two of the resources and could not be expressed in another lymphocyte type (e.g., an NK or CD4^{+} or CD8^{+} T-cell). A FAIME score was then calculated for each tumor sample, for each cell type, by producing a weighted ranking of the genes in each sample and then determining the ranking of the marker genes for a particular cell type as compared to the genes not associated with that cell type. Finally, a student's t-test was used to assess whether the FAIME scores for a particular cell type were significantly different (q-value <0.05) between HPV(+) and HPV(−) tumors. In a separate group level assessment, the marker gene sets for each tissue and cell type significantly over-represented for DEGs (Bonferroni corrected p-value <0.05) were identified using a hypergeometric test.

REFERENCES

1. Marsh D, Suchak K, Moutasim KA, Vallath S, Hopper C, Jerjes W, Upile T, Kalavrezos N, Violette SM, Weinreb PH, Chester KA, Chana JS, Marshall JF, Hart IR, Hackshaw AK, Piper K, et al. Stromal features are predictive of disease mortality in oral cancer patients. J Pathol. 2011; 223:470-481.
2. Trapnell C, Pachter L and Salzberg SL. TopHat: discovering splice junctions with RNA-Seq. Bioinformatics. 2009; 25:1105-1111.
3. Anders S, Pyl PT and Huber W. HTSeq-a Python framework to work with high-throughput sequencing data. Bioinformatics. 2014.
4. Nikolayeva O and Robinson MD. edgeR for differential RNA-seq and ChIP-seq analysis: an application to stem cell biology. Methods in molecular biology. 2014; 1150:45-79.
5. Robinson MD, McCarthy DJ and Smyth GK. edgeR: a Bioconductor package for differential expression analysis of digital gene expression data. Bioinformatics. 2010; 26:139-140.
6. Robinson MD and Oshlack A. A scaling normalization method for differential expression analysis of RNA-seq data. Genome biology. 2010; 11:R25.
7. Livak KJ and Schmittgen TD. Analysis of relative gene expression data using real-time quantitative PCR and the 2(-Delta Delta C(T)) Method. Methods. 2001; 25:402-408.
8. Bustin SA, Benes V, Garson JA, Hellemans J, Huggett J, Kubista M, Mueller R, Nolan T, Pfaffl MW, Shipley GL, Vandesompele J and Wittwer CT. The MIQE guidelines:

minimum information for publication of quantitative real-time PCR experiments. Clinical chemistry. 2009; 55:611-622.

9. Yang X, Regan K, Huang Y, Zhang Q, Li J, Seiwert TY, Cohen EE, Xing HR and Lussier YA. Single sample expression-anchored mechanisms predict survival in head and neck cancer. PLoS computational biology. 2012; 8:e1002350.
10. Shoemaker JE, Lopes TJ, Ghosh S, Matsuoka Y, Kawaoka Y and Kitano H. CTen: a web-based platform for identifying enriched cell types from heterogeneous microarray data. BMC genomics. 2012; 13:460.
11. Abbas AR, Baldwin D, Ma Y, Ouyang W, Gurney A, Martin F, Fong S, van Lookeren Campagne M, Godowski P, Williams PM, Chan AC and Clark HF. Immune response in silico (IRIS): immune-specific genes identified from a compendium of microarray expression data. Genes Immun. 2005; 6:319-331.
12. Watkins NA, Gusnanto A, de Bono B, De S, Miranda-Saavedra D, Hardie DL, Angenent WG, Attwood AP, Ellis PD, Erber W, Foad NS, Garner SF, Isacke CM, Jolley J, Koch K, Macaulay IC, et al. A HaemAtlas: characterizing gene expression in differentiated human blood cells. Blood. 2009; 113:e1-9.
13. Palmer C, Diehn M, Alizadeh AA and Brown PO. Cell-type specific gene expression profiles of leukocytes in human peripheral blood. BMC genomics. 2006; 7:115.
14. Grigoryev YA, Kurian SM, Avnur Z, Borie D, Deng J, Campbell D, Sung J, Nikolcheva T, Quinn A, Schulman H, Peng SL, Schaffer R, Fisher J, Mondala T, Head S, Flechner SM, et al. Deconvoluting post-transplant immunity: cell subset-specific mapping reveals pathways for activation and expansion of memory T, monocytes and B cells. PloS one. 2010; 5:e13358.
15. Whitney AR, Diehn M, Popper SJ, Alizadeh AA, Boldrick JC, Relman DA and Brown PO. Individuality and variation in gene expression patterns in human blood. Proceedings of the National Academy of Sciences of the United States of America. 2003; 100:1896-1901.

SUPPLEMENTARY FIGURES

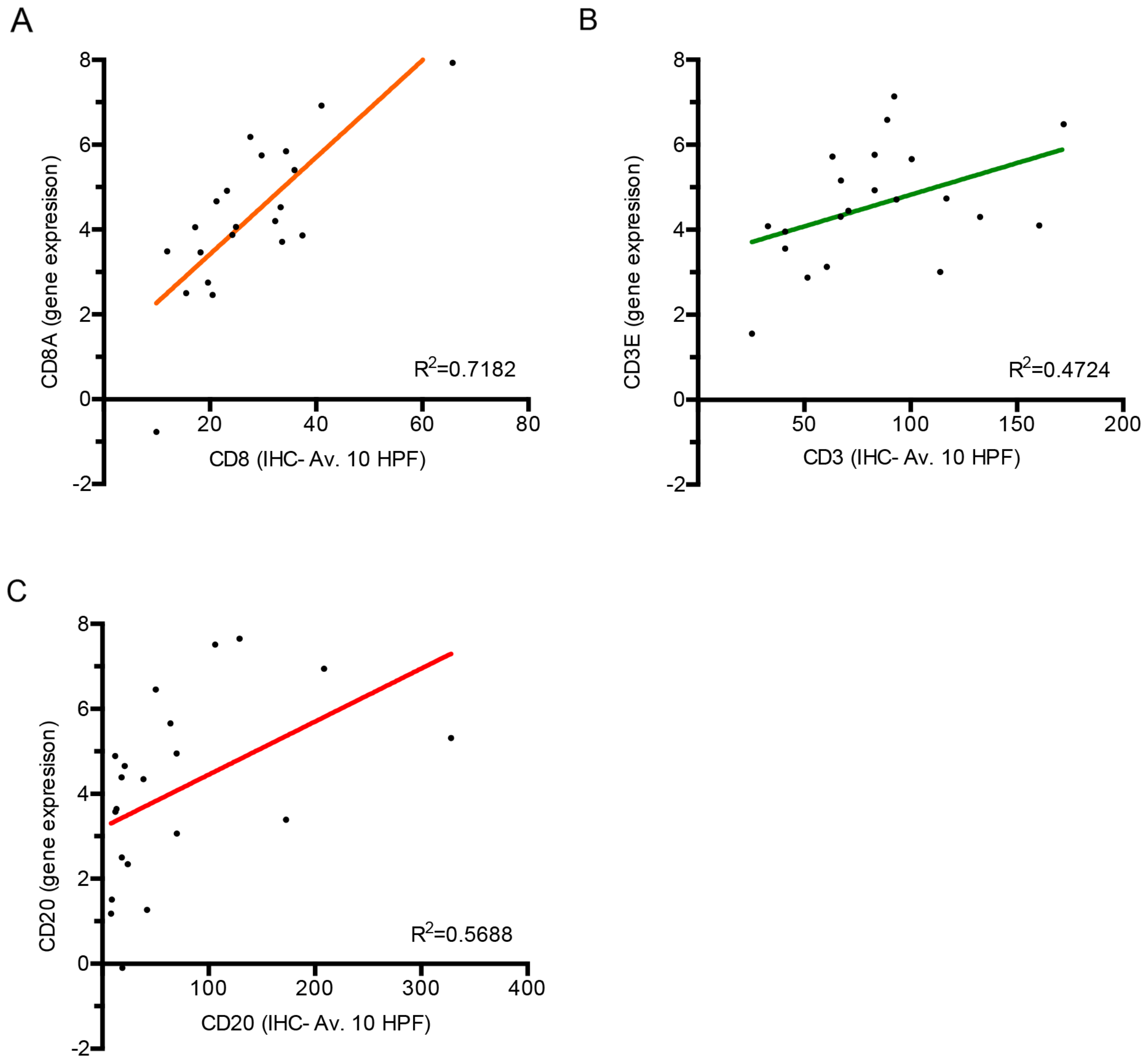

Supplementary Figure S1: Spearman correlation analysis between immunohistochemistry and gene expression for Immune cell types. Correlation of IHC and gene expression determined by RNA-Seq for **A.** CD8, **B.** CD3 and **C.** CD19/CD20 B-cells. A positive correlation was observed for each marker when counting 10 high power fields and correlating it with the level of immune gene transcripts determined by RNA-Seq.

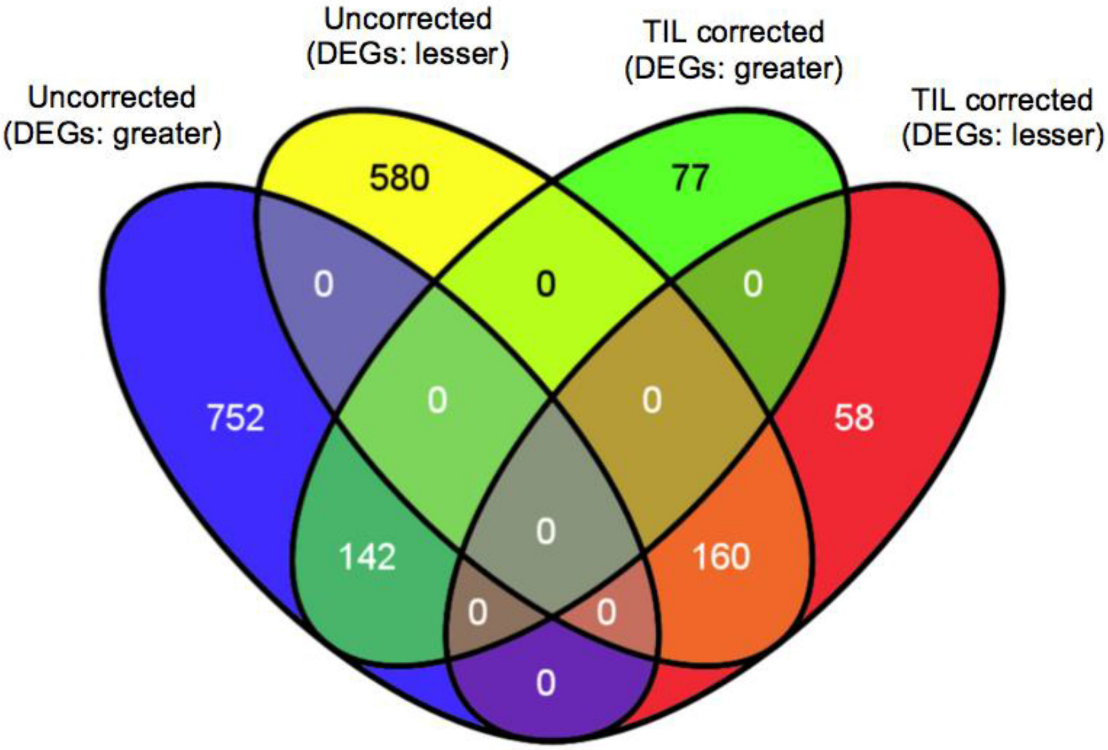

Supplementary Figure S2: Differentially expressed genes between HPV(+) and HPV(−) tumors; overlap of DEGs identified from uncorrected and TIL corrected gene expression data. A Venn diagram to illustrate the overlap of DEGs between HPV(+) and HPV(−) tumors identified directly by RNA-Seq (n=1,634) or following correction of the gene expression data to account for numbers of infiltrating immune cells (n=467); genes expressed to a greater or lesser extent in HPV(+) versus HPV(−) tumors. For TIL corrected gene expression data, new DEGs were identified, which were expressed to a greater (n=77) or lesser extent (n=58) in HPV(+) tumors.

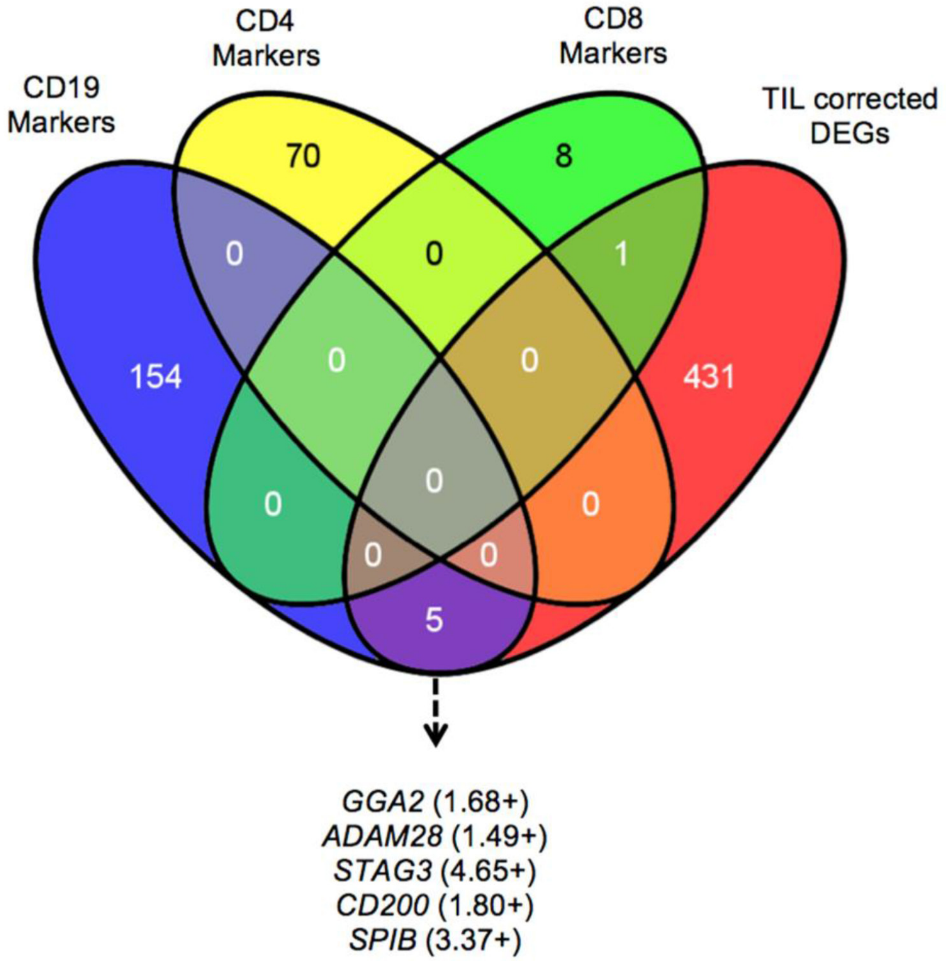

Supplementary Figure S3: Differentially expressed genes between HPV(+) and HPV(−) tumors identified from TIL corrected data; overlap of DEGs expressed to a greater extent in HPV(+) tumors with published immune cell type gene sets. A Venn diagram to illustrate the overlap of 437 DEGs expressed to a greater extent in HPV(+) versus HPV(−) tumors identified from TIL corrected gene expression data with immune cell type-specific marker genes as defined by at least two published databases: CD19 markers (n=159), CD4 markers (n=70) and CD8 markers (n=9). An increase in the expression of 5 B-cell-associated genes was observed in HPV(+) compared to HPV(−) tumors: *GGA2, ADAM28, STAG3, CD200* and *SIPB*; fold-change in expression is shown in *brackets*.

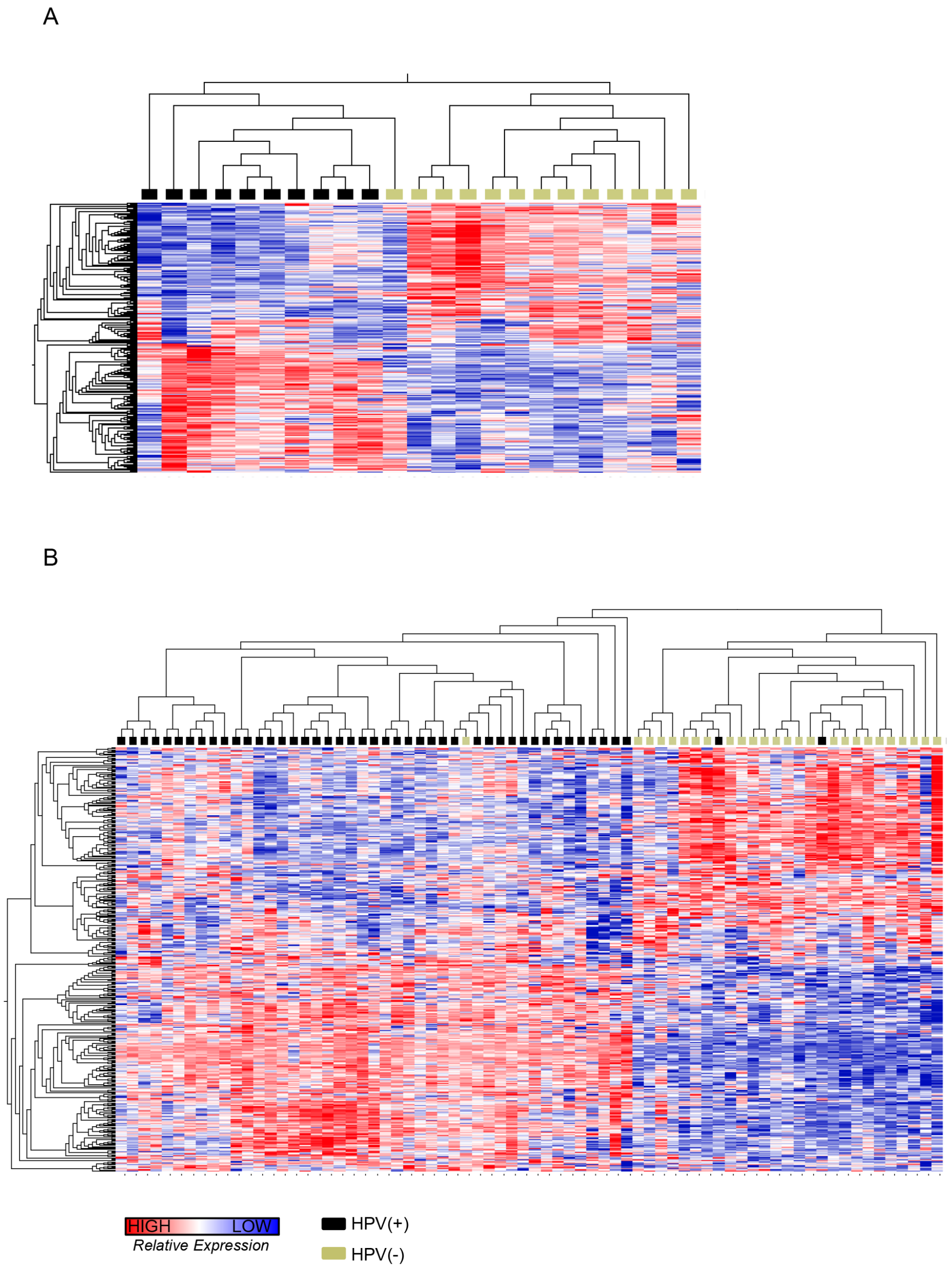

Supplementary Figure S4: Expression of TIL corrected DEGs in an independent sample cohort. Heatmaps* to illustrate gene expression of the TIL corrected DEGs (n=437) between HPV(+) and (−) tumors. **A.** a heatmap of our HNSCC dataset (HPV(+) n=10 and HPV(−) n=13). **B.** a heatmap of the TCGA HNSCC dataset (HPV(+) n=46 and HPV(−) n=26); publically available data from anatomically matched tumors arising in the oropharynx, tonsil and base of tongue. In both datasets, tumors cluster according to HPV status; sub-clusters are evident in the larger TCGA dataset. *Unsupervised clustering of gene expression data was normalized using the TMM method followed by variance stabilizing transformation of the TMM normalized data. Each row represents normalized gene expression values for a given gene; each column represents the gene expression for a given tumor: red shading denotes greater gene expression, blue shading denotes lower gene expression. Hierarchical clustering of genes and tumors based on their expression profile is reflected in the dendrograms to the left and the top of the heatmap, respectively, and was performed by calculating distance using the Pearson's correlation metric and then clustering distance using the ward linkage method.

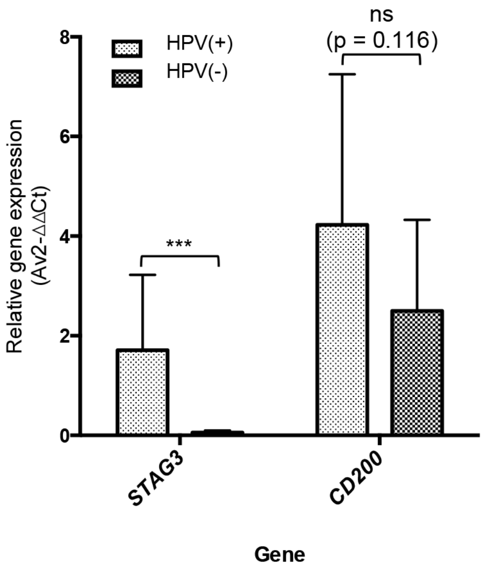

Supplementary Figure S5: Confirmation of RNA-Seq data by RT-qPCR of *STAG3* and *CD200*. The average relative gene expression of *CD200* and *STAG3* was measured by RT-qPCR* in RNA extracted from the whole tumor, as used for the RNA-Seq analysis. The expression of *STAG3* and *CD200* was determined for HPV(+) (n=8) and HPV(−) (n=8) tumors. This showed the same trend with HPV(+) tumors compared to HPV(−) tumors having increased expression of *STAG3* and *CD200* (S*TAG3*, ***p<0.001 and *CD200* nsd, p=0.116). *Relative gene expression by RT-qPCR, calculated using the comparative Ct method with *Actin* as the control gene (2-ΔΔCt method) (23). *Asterisks* in column labels indicate a significance level of a two-sample *t*-test comparison of RT-qPCR between HPV(+) and HPV(−) tumors: ns = not significant (value stated) and ****P* <0.001).

Supplementary Table S1: A list of differentially expressed genes between HPV(+) and HPV(-) tumors identified by RNA-Seq analysis.

See Supplementary File 1

Supplementary Table S2: Gene ontology analysis of DEGs expressed to a geater extent in HPV(+) vs HPV(-) tumors.

See Supplementary File 2

Supplementary Table S3: Gene ontology analysis of DEGs expressed to a lesser extent in HPV(+) vs HPV(-) tumors.

See Supplementary File 3

Supplementary Table S4: Pathway analysis of DEGs expressed to a geater extent in HPV(+) vs HPV(-) tumors.

See Supplementary File 4

Supplementary Table S5: Pathway analysis of DEGs expressed to a lesser extent in HPV(+) vs HPV(-) tumors.

See Supplementary File 5

Supplementary Table S6: Marker gene sets whose expression was associated with the different lymphocyte cell subsets (B-cells, NK cells and CD4+ and CD8+ T-cells).

See Supplementary File 6

Supplementary Table S7: A list of differentially expressed genes between HPV(+) and HPV(-) tumors identified by RNA-Seq analysis followed by correction for TIL number.

See Supplementary File 7

Supplementary Table S8: Gene ontology analysis of DEGs expressed to a geater extent in HPV(+) vs HPV(-) tumors (TIL corrected data).

See Supplementary File 8

Supplementary Table S9: Gene ontology analysis of DEGs expressed to a lesser extent in HPV(+) vs HPV(-) tumors (TIL corrected data).

See Supplementary File 9

Supplementary Table S10: Pathway analysis of DEGs expressed to a geater extent in HPV(+) vs HPV(-) tumors (TIL corrected data).

See Supplementary File 10

Supplementary Table S11: Pathway analysis of DEGs expressed to a lesser extent in HPV(+) vs HPV(-) tumors (TIL corrected data).

See Supplementary File 11
